# Making the case for a resurgent U.S. independent veterinary practice segment: a SWOT analysis

**DOI:** 10.3389/fvets.2025.1558745

**Published:** 2025-05-13

**Authors:** Martin Traub-Werner, Matthew Salois, Charlotte McKay, Marne Platt

**Affiliations:** ^1^Vetbooks, Arlington, TX, United States; ^2^Veterinary Management Groups, Johns Creek, GA, United States; ^3^Fundamental Capabilities, Fort Lauderdale, FL, United States

**Keywords:** veterinary practice, veterinary industry, practice owner, corporate veterinary practice, veterinary ecosystem, independent veterinarian, veterinary business

## Abstract

Private equity and corporate ownership of veterinary practices has largely been characterized as either wholly good or wholly bad. Supporters point to increased returns and access to capital and investments in associates, technology, and other resources. Opponents point to unnecessary debt and decreased quality of care. Good or bad, they are part of the practice landscape. This SWOT (Strengths, Weaknesses, Opportunities, and Threats) analysis of independent veterinary practice reveals that, while corporate veterinary practices have a role, a thriving independent practice segment still provides a deeply satisfying career and a viable living. We conclude that, with the current economic conditions and the appropriate modifications in the veterinary ecosystem, conditions are ripe for a resurgence of independent veterinary practice ownership.

## 1 Introduction

Corporate practices, consolidators, and private equity are words that became part of our conversation about the future of veterinary medicine more than 30 years ago, with VCA’s first clinic purchase (1). Veterinary medicine was once and first a world of independent businesses: practices owned by one or a few people. A wave of consolidation has driven the increase of private equity in veterinary medicine over the last few decades, and not just in the United States (2–6).

In the beginning, consolidators saw independent veterinary practices as undervalued, underpriced bargains (7). The economics of veterinary medicine are attractive: Practices are profitable. Veterinary services are seen as more resilient in economic downturns, delivering consistent returns even through a volatile business cycle (7). Pet owners will pay for pet care, even in tough times. That, combined with the unusually low cost of capital over the past few decades, created the conditions for rapid market consolidation (8). Consolidators quickly built a large market presence in veterinary medicine. Since those first corporate entrants in the 1980s, private equity or venture capital investors, private companies formed by individuals pooling their money to invest, and publicly traded firms have all purchased independent veterinary practices (9–11). Most corporate practices or consolidators today are backed by private equity (9). Private equity groups are not publicly traded and usually look for existing businesses that can be improved with efficiencies and experienced management, then sold for profit (12). In most cases, these businesses are owned by non-veterinarians, generally by individuals who come from outside the veterinary industry. By 2017, corporate practices represented approximately 10% of general companion animal practices (13). Today, approximately 75% of specialty and emergency practices and 25% of primary care practices, representing 50% of nationwide veterinary revenues, are owned by corporate consolidators (14).

Corporate veterinary medicine is not for everyone. While the economic conditions for corporate consolidators have been attractive, capital and interest rates are rising, and current economic conditions are slowing the rate of corporate expansion into veterinary medicine (15). Independent practices have an opportunity to regain their foothold in the industry. What will help independent practices thrive? In this paper we evaluate the strengths, weaknesses, opportunities, and threats facing independent veterinary practices using SWOT analysis.

## 2 SWOT analysis: independent veterinary practice

**Figure 1:**
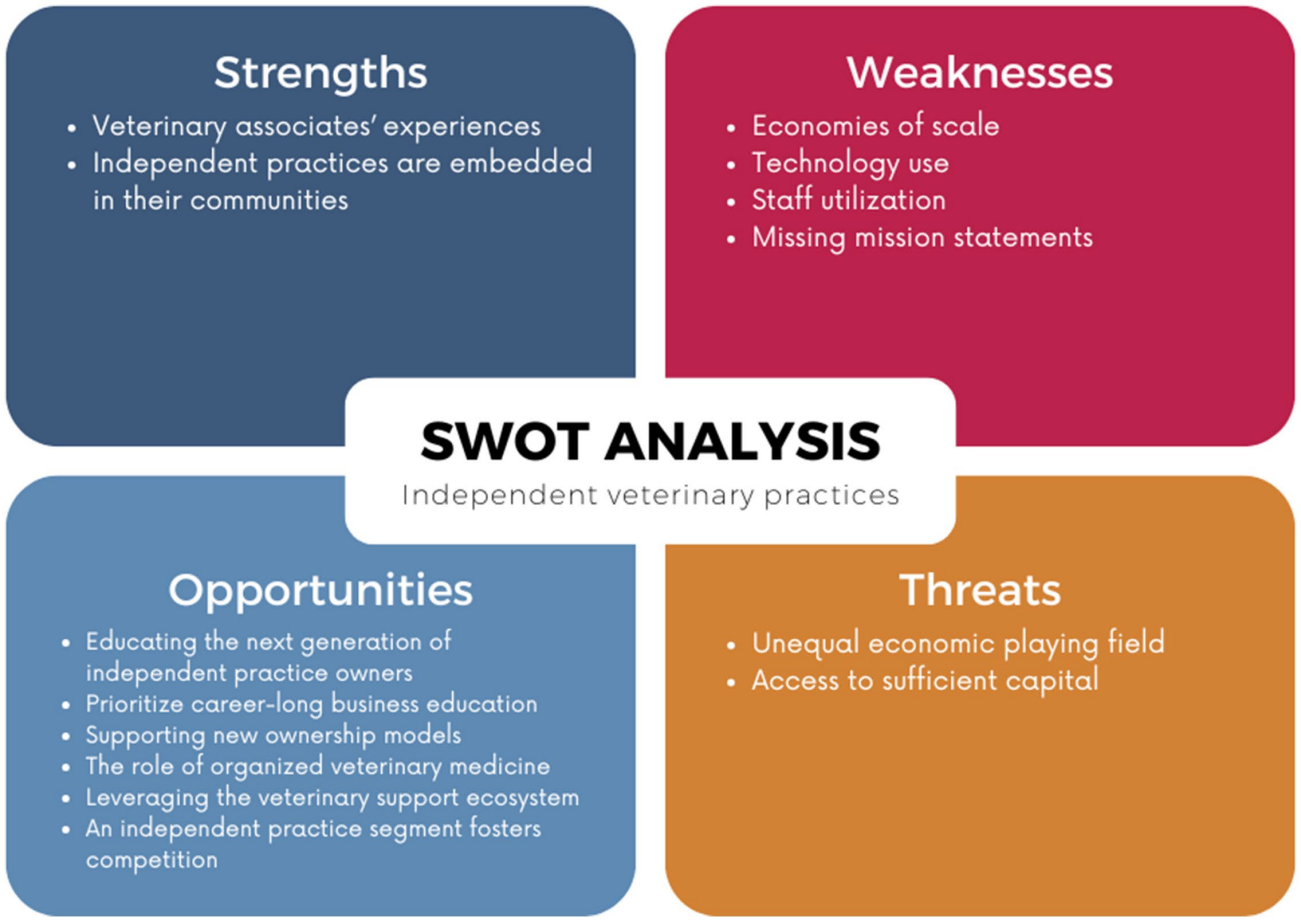
SWOT analysis of the independent veterinary practice segment.

SWOT analysis is a tool for evaluating businesses and projects in relation to competitors that has been in use since at least 1960 (16). We believe that too much emphasis has been placed on corporate practice characteristics and have elected to conduct a strategic evaluation of the characteristics of the independent veterinary practice segment: its internal strengths and weaknesses and external opportunities and threats. We describe the independent practice segment’s advantages and disadvantages in relation to corporate practices and identify conditions within the broader veterinary ecosystem that could benefit or negatively affect an independent practice (16). This paper provides a more structured analysis of common perceptions of the advantages and limitations of independent veterinary practice ownership (Figure 1). Results of this SWOT analysis demonstrate that independent practice ownership still provides a deeply satisfying career and viable living, that independent veterinary practices can successfully compete with corporate practices, and that the current economic conditions are ripe for a resurgence of independent veterinary practice ownership.

### 2.1 Strengths

#### 2.1.1 Veterinary associates’ experiences

Veterinary associates generally report better work experiences in independent practices. In a recent study, a majority of associates (55.1%) preferred working in independent practice, even though approximately 70% of those associates responding were employed in corporate practice (17). Veterinarians in corporate practices reported feeling significantly greater pressure to see more clients per shift and generate more revenue (17). Although insurance, continuing education, and mental wellness programs were significantly more common in corporate practice, associates in independent practice were significantly more satisfied with practice administration, mentorship, and culture (17). Associates in independent veterinary practices were also more satisfied with compensation and team turnover rates (17). Those working in independent practices owned by one veterinarian were more likely to stay for the next 5 years than were associates working in corporate practices (18). “Voting with one’s feet” by finding another position elsewhere is an unambiguous indicator of overall job satisfaction.

Senior veterinary staff in independent practices were also more satisfied. While 21% of corporate medical directors experienced “high” or “very high” levels of burnout, independent practice owners were the group most likely to report low levels of burnout (19–22). Independent practice owners have also reported high satisfaction rates with their jobs (84%), lifestyles (76%), and compensation (75%) (19).

#### 2.1.2 Independent practices are embedded in their communities

Independent veterinary practices that are embedded in and supported by their business ecosystem give back to it in turn. While corporations must satisfy their shareholders’ profit expectations, independent veterinary practices have more flexibility and incentive to reinvest profits in their local communities (23). An independent practice owner is more likely than a remote corporate regional or national director to know other local business owners through local civic organizations or as part of their social network. When the time comes to support a local community initiative or partner with another business, the independent practice owner may be more likely to choose another independent local business owned by someone within their network. Doing so strengthens local relationships, builds practice equity, and binds the practice and the community together. This makes economic as well as social sense: According to the 2022 Small Business Saturday Consumer Insights Survey, “$0.68 of every dollar spent at a small business in the U.S. stays in the local community…every dollar spent at small businesses creates an additional $0.48 in local business activity as a result of employees and local businesses purchasing local goods and services” (24). Corporate practices, by contrast, may be more likely to develop nationwide, “headquarters-to-headquarters” partnerships that have fewer local network benefits than those between small businesses in the same ecosystem (25, 26).

### 2.2 Weaknesses

#### 2.2.1 Economies of scale

One gap between independent practices and corporations is in economies of scale (12, 27, 28). Large veterinary corporations can decrease some costs by consolidating back-office work and negotiating volume discounts. Many corporate veterinarians appreciate not having to handle all the paperwork that comes with ownership. Fortunately, independent veterinarians have options to help them gain economies while remaining independent: Buying groups give members access to discounts from major suppliers. New service providers, many of which also start as small independent business, are available to provide support for the back-office and administrative work, including client reminders, online pharmacy fulfillment services, human resources, and training (29). Technology and artificial intelligence can also fill the gap, for example with online scheduling, chatbots for answering simple client questions, and voice-to-text transcription.

#### 2.2.2 Technology use

Appropriate use of technology improves efficiency, operations and client communications for many businesses, and corporate practices can use their back-office teams for implementation without taking away from client-focused activities. Independent veterinary practices are falling behind. Only 41% of practice owners are enthusiastic about the use of new technologies in their practice (30). Equine and mixed animal private practices were the highest adopters of technology (59.0% and 41.0% respectively), while only 36.4% of companion animal and 31.6% of food animal private practices were using new technology to improve their practices (21). Among practice owners who felt they were lagging in using new technologies, 17.2% were simply uninterested; 31.3% blamed a lack of time, and 26.3% found it too expensive (30). While 80% of companion animal exclusive practices use Practice Information Management Software (PIMS), barely two-thirds use Electronic Medical Record Systems (EMRS) or an online pharmacy, 38.6% offer online appointment scheduling, and 25.7% offer telehealth (21). Private equine practice numbers revealed different gaps: 67.7% use PIMS, 74.2% use an EMRS, and 50% offer an online pharmacy, while 37.1% use telehealth and only 9.7% offer online scheduling (21). Independent practices must embrace technology’s advantages to remain competitive. Actively promoting their own online appointment scheduling and home delivery platforms and strategic use of telehealth, for example, will help these practices reduce costs and increase efficiency and profit, while potentially improving client convenience and satisfaction.

#### 2.2.3 Staff utilization

Utilizing the right non-veterinarian staff in any veterinary practice has been shown to increase gross revenue and increase practice efficiency (31–34). In 2023, only 56% of veterinary practices had practice managers, requiring medical staff to handle more non-medical work (19). Veterinary practice managers allow practice owners more time to focus on clinical, revenue-generating activities. Credentialed veterinary technicians (CVTs) are similarly underutilized, leading to job dissatisfaction, burnout, practice inefficiency and high staff turnover rates (35, 36). Independent veterinary practices that make effective use of every staff member’s skills, and shift non-medical tasks away from medical staff, are more productive than those that do not (35). Practices that fail to assign work to the most appropriate team members undermine their own success and their ability to compete with more efficient corporate practices. Closing this gap is within the independent practice owner’s control.

Improved staff utilization is the counterpoint to the debate over the need for educating new veterinarians and introducing a mid-level practitioner (37, 38). We contend that with smarter utilization of existing staff, emerging technologies, and strategic partnerships, the profession can meet future demand without relying on mid-level practitioners or expanding the number of veterinary schools. Even as the supply of veterinarians increases with larger class sizes at existing schools and more than a dozen new veterinary schools pursuing accreditation, patient visits are down and AI-driven productivity is on the rise (15, 39, 40). Recent analysis that considers market uncertainty and careful model selection has determined that the currently planned supply of veterinarians will be sufficient (41). Introducing mid-level practitioners into this environment could further strain earning potential for veterinarians, credentialed veterinary technicians (CVTs), and mid-levels themselves, making it more difficult to repay educational debt or earn a sustainable wage. Rather than waiting for an increased supply of veterinarians or a new type of non-veterinarian staff, independent practice owners should focus on what they can directly control today: optimizing their existing workforce, allowing all staff members to work at the top of their qualifications and embracing innovation to provide quality care.

#### 2.2.4 Missing mission statements

There is a growing divide between the goals of private equity groups that own veterinary practices and the fundamental purpose that many veterinary professionals see as their “North Star.” Most independent practice owners would not say that profitability is their number one goal, even though they acknowledge its importance. Every business must make a profit to pay its employees and suppliers; a business is not sustainable if it is not profitable. Although private veterinary practice is no different in this regard, it is also strongly purpose driven. That purpose is to keep animals happy and healthy and provide the best possible quality of care, for the benefit of society (42).

A mission statement lays out an organization’s purpose and objective, providing direction for an organization’s operations. Corporations with well-defined mission statements, whether emphasizing care or profit, can better drive employee behavior to align with their strategic goals. Organizations whose employees’ activities align with their mission statement show a direct relationship with financial performance (43). Mission statements of private equity companies backing corporate practices emphasize profit, generating returns, and maximizing value. For example, JAB Consumer Partner’s mission statement emphasizes “*creating sustainable compounding returns*” (44). The mission statements of purpose-driven practices emphasize pets and health care, for example, “*Improving the health and quality of life of every animal we touch*” (45). However 46.7% of independent practices do not have a vision or mission statement, while 10.9% have the statement but do not share it with employees (20). Independent practice owners should refine and publicize their mission statements, making their goals clear to employees and clients. Sharing and living that mission every day with every client can motivate staff and improve practice performance.

### 2.3 Opportunities

While independent veterinary practices face internal challenges, external opportunities exist as well. Grasping these opportunities can help the independent practice segment to thrive.

#### 2.3.1 Educating the next generation of independent practice owners

A robust independent veterinary practice segment requires a pipeline of motivated new and future practice owners. Education is the first step. Veterinary schools should encourage students who want to own practices by providing training in critical veterinary-specific business and leadership skills. Many schools host chapters of the Veterinary Business Management Association^1^; some veterinary schools have introduced formal training programs (46). More are needed.

#### 2.3.2 Prioritize career-long business education

Veterinary medicine already provides opportunities for life-long learning for medical skills. The same must be available for future independent practice owners. Associate veterinarians interested in practice ownership must speak up, understanding when and how to ask about ownership possibilities. They must hone their skills in finance, business management, and leadership to be ready when their time for ownership comes. Even experienced practice owners can benefit from peer support groups and ongoing education in business management and operations (47). However, 82% of private practice owners said they do not provide any financial training to their staff (48). Practice owners who are serious about selling to an associate must invest in educating and mentoring their associates. This training must begin years in advance of an actual sale. Newly graduated veterinarians are already interested in mentoring for clinical skills; they also need willing mentors in practice ownership and leadership (49–52). Creating the next generation of independent veterinary practice owners requires formal programs, informal networks, and continuing education opportunities today.

#### 2.3.3 Supporting new ownership models

The veterinary profession needs new ownership models that can support licensed technicians and certified practice managers in becoming full or partial practice owners. Their passion for the industry and ability to manage a business are no less than that of veterinarians. This will require careful negotiation with states that currently restrict practice ownership to veterinarians. Employee Stock Ownership Programs (ESOP) enable employees to own portions or the entirety of a business. A few veterinary practices groups, like Suveto, use this approach (53).

#### 2.3.4 The role of organized veterinary medicine

The AVMA and consulting groups like VetPartners^2^ must make independent practice ownership a priority, by working with buyers and sellers of veterinary practices to demystify the financial aspects and providing ongoing training and expert support. The AVMA could be a key player in negotiating changes to state laws regulating veterinary practice ownership, so that certified veterinary staff can become practice owners. The Independent Veterinary Practitioners Association (IVPA) is already working in this area (54). Formal recognition of the IVPA by the AVMA’s House of Delegates would bring additional credibility and momentum toward advocating for independent practices.

#### 2.3.5 Leveraging the veterinary support ecosystem

Corporations can consolidate back-office duties such as human resources, marketing, accounting, customer support, and payroll across multiple practices, share staff across practices, make bulk purchases, and receive rebates from suppliers through bundle purchasing (26). Independent veterinary practice owners can take advantage of advances in business and technology to gain many of the benefits expected from corporatization. Buying groups and other member-based organizations give smaller practices greater purchasing power (55). Simple online searches provide a range of complementary providers: Legal, HR, and recruitment firms specializing in veterinary medicine provide access to expertise that many veterinarians do not have. Other specialty businesses support independent veterinary practices for accounting, marketing, medical recordkeeping, scheduling, pharmacy delivery, and even mentoring. On the medical side, teletriage, pet-wearable devices, and remote specialist consultations are widely available or on the verge of becoming so. These give even the most remote practices and patients access to the latest medical advances. Manufacturers and suppliers of goods and services can support new independent veterinary practices with special programs to help them get off the ground, including introductory discounts, special payment terms, and marketing support.

#### 2.3.6 An independent practice segment fosters competition

Most markets benefit from robust competition (22, 56). Consolidation in the veterinary industry has caught the attention of the Federal Trade Commission (FTC). Worried about competitiveness in local markets, they have begun intervening in large-scale mergers and acquisitions, requiring at least one firm that owned approximately 1,500 practices to divest some of them and obtain FTC permission before acquiring more (57). The FTC understands that competition is the seed of innovation, creativity and consumer choice. The consequence of reduced competition, most notably through price increases, can be seen in many industries, including pharmaceuticals, broadband, mobile telephony, personal banking, healthcare, insurance, air travel, and even beer (58). Underinvestment in R&D, lack of consumer choice, and poor customer service are other recognized negative effects of reduced competition (59, 60). The negative effects of reduced competition in the veterinary industry impact veterinarians, staff, patients, and pet families. A thriving independent segment encourages creative problem solving, new ways of serving clients, and new adjacent businesses to support those independent practices.

### 2.4 Threats

#### 2.4.1 Unequal economic playing field

Consolidators with large numbers of practices can exert their market power to obtain lower prices from manufacturers, distributors, and other suppliers (12, 26, 27). They then attract pet owners by advertising ever-lower retail prices. This creates a race to the bottom among suppliers with no long-term benefits, by furthering consolidation across the whole veterinary industry, not just within veterinary practices. Moreover, squeezing suppliers’ profit margins can decrease innovation and investment in research and development (61). Minimum Advertised Pricing (MAP), in which the manufacturer determines the lowest price at which a seller should advertise the product, plays a key role in dampening this negative impact by leveling the playing field between, for example, independent practices and large online pharmacies.

#### 2.4.2 Access to sufficient capital

New practice owners must have access to the capital they need to buy or open new independent practices. In a Veterinary Management Groups survey of former members who sold to corporate practices, 47% of former practice owners wanted to take advantage of high multiples (62). On the other hand, 71% of owners who had sold their practice to a corporation would have preferred to sell to an associate veterinarian; 59% reported that the associate did not have the economic ability to buy the practice (62). Excessively high multiples only worsen this problem. Associates interested in practice ownership cannot compete with the high multiples that private equity groups have been offering for the purchase of a private practice. Fortunately, these high multiples have been decreasing since 2022 (63).

The inability to acquire and deploy capital at the level of corporate and private equity-backed practices may be the biggest threat to future independent practices. By its very nature, private equity is an accumulation of capital intended to be deployed and generate a return. PE-backed practices bring in large pools of almost entirely unregulated capital to facilitate their growth and expansion (64, 65). Meanwhile, the typical independent veterinarian is subject to the tightly regulated traditional lending market, with its restrictions on capital access and deployment (66). Today’s rising interest rates add another hurdle. This is a fundamental challenge for all small businesses, not just independent veterinary practices.

However, the rise in interest rates also poses problems for private equity. The private equity business model depends on low interest rates, which allow them to put cash to work for little cost so they can grow and then sell the business (10). Higher interest rates threaten their underlying business model, forcing them to focus more on operations in the practices they purchase to find growth. Since private equity owners are not experts in veterinary medicines or operating veterinary hospitals, this requires more attention and effort, costing time and money and making veterinary acquisitions less attractive. Independent veterinary practice owners always pay attention to practice operations, so while high interest rates are certainly a challenge, they do not disrupt an independent practice’s basic business model in the same way. While some independent practices grow by expanding and acquiring additional hospitals, most focus on optimizing revenue per patient and improving cost efficiency—growth through margin rather than scale. This strategy is reflected in benchmark studies of well-managed practices (67).

In the short term, the current rise in interest rates is a problem for both independent and corporate practices, moreso for the latter. In the long run, when interest rates decrease, private equity will once again have access to cheap capital. Independent veterinarians, still at the mercy of traditional lenders, will again be at a disadvantage unless new ownership models or easier ways for them to access capital develop.

## 3 Conclusion

Corporate veterinary medicine is not for everyone. Many associates would prefer to work for independent veterinary practices, citing among other aspects better mentoring and more attractive practice cultures. Embedded in their local social and business ecosystem, independent veterinary practices contribute to a competitive market and a robust community, while providing personalized, compassionate care. What will help independent veterinary practices grow and thrive in the industry?

Building strong independent practices begins in school and continues throughout a veterinarian’s career, involving many parts of the veterinary ecosystem. Schools, continuing educators, and professional organizations must supply current and future practice owners with the financial and leadership training required throughout their careers. Organized veterinary medicine must strongly advocate in favor of the needs of independent practice owners. Suppliers should offer incentive programs for independent practices, much as they do for corporate practices. Independent practice owners must actively pursue the next generation of practice owners through mentorship and discussion about practice ownership, well in advance of any actual practice sale. Today’s independent practice owners must leverage their unique advantages: close ties in their communities, a powerful emotional mission, and the ability to quickly respond to local needs. New technologies, service providers specifically for veterinary medicine, appropriate staff utilization, and support from peer groups can help independent practice owners become more efficient, freeing their time and energy for the practice of medicine and the close client and community relationships that underlie their competitive advantage.

The strengths, weaknesses, opportunities, and threats identified in this SWOT analysis of independent veterinary practices highlight possibilities for those practices to better compete with corporate practices. The rising cost of capital, while challenging for independent practice owners, will also slow the rate of corporate expansion. Cultivating and nourishing a sustainable legacy of independently owned veterinary practices is good for competition, for veterinarians and staff, and ultimately for pets and society. Encouraging and supporting interested veterinarians to become independent practice owners is essential for the future of veterinary practice.

## Data Availability

The original contributions presented in the study are included in the article/supplementary material, further inquiries can be directed to the corresponding author.

## Footnotes

^1^ https://vbma.biz/chapters/

^2^ https://vetpartners.org/?

## References

[1] VCA. Our story. (2024). Available online at: https://vcahospitals.com/about-us (Accessed 24 January 2024).

[2] Gärtner MC. *Private equity challenge–capital structure* [Master’s thesis, Universidade NOVA de Lisboa (Portugal)] (2023).

[3] Reader D Summers S. Paws for thought: Putting UK vet acquisitions on a tightened leash *CPI antitrust chronicle*. (2024).

[4] BizJournal. Ares Management to sell National Veterinary Associates L.a. Business First (2019). Available online at: https://www.bizjournals.com/losangeles/news/2019/06/21/ares-management-to-sell-national-veterinary.html (Accessed October 24, 2024).

[5] Inflexion. Inflexion partners with TPP founders to support further growth. (2024). Available online at: https://www.inflexion.com/news-insights-events/press-releases/2024/inflexion-partners-with-tpp-founders-to-support-further-growth/ (Accessed October 24, 2024).

[6] Wright M Gilligan J Amess K. The economic impact of private equity: what we know and what we would like to know. Ventur Cap. (2009) 11:1–21. doi: 10.1080/13691060802151887

[7] Ackerman Group. Why are so many veterinary hospitals selling to corporate? (2022). Available online at: https://ackerman-group.com/learning-center/why-are-so-many-veterinary-hospitals-selling-to-corporate/ (Accessed April 5, 2024).

[8] Federal Reserve Bank of Cleveland. 10-year real interest rate 1 Jan 1982–2023. (2023). Available online at: https://fred.stlouisfed.org/series/REAINTRATREARAT1YE (Accessed 20 January 2024).

[9] Ackerman Group. Revealed: How private equity is reshaping veterinary care. (2024). Available online at: https://ackerman-group.com/current-market/private-equity-who-are-these-people/ (Accessed October 22, 2024).

[10] Martin R. Harvard Business Review. It’s time to replace the public corporation. (2021). Available online at: https://hbr.org/2021/01/its-time-to-replace-the-public-corporation (Accessed October 23, 2024).

[11] Veterinary Integration Solutions. Veterinary consolidators: North American market analysis. (2022) Available online at: https://vetintegrations.com/insights/veterinary-consolidators/ (Accessed February 10, 2024).

[12] MedPac. Report to congress: Medicare and the health care delivery system. Washington D.C.: MedPac (2021).

[13] Nolen RS. The corporatization of veterinary medicine. J Am Vet Med Assoc. (2018) 253:1376–9.

[14] Brakke Consulting. 2022 Industry Overview. Orlando, Florida: Brakke Consulting (2022).

[15] Brakke Consulting. 2025 Industry overview. Las Vegas, Nevada: WVC (2025).

[16] Teoli D Sanvictores T An J. SWOT Analysis. StatPearls Treasure Island (FL): StatPearls Publishing (2023). 30725987

[17] Kogan LR Rishniw M. Differences in perceptions and satisfaction exist among veterinarians employed at corporate versus privately owned veterinary clinics. J Am Vet Med Assoc. (2023) 261:1838–46. doi: 10.2460/javma.23.06.0326, PMID: 37607680

[18] McKay CH Vaisman JM. Psychological safety, purpose, path, and partnership reduce associate veterinarian desire to leave current employment. J Am Vet Med Assoc. (2023) 1:1–7. doi: 10.2460/javma.23.03.0158 37380161

[19] American Veterinary Medical Association, 2023 AVMA report on the economic state of the veterinary profession, (2023) Schaumburg, IL.

[20] Merck Animal Health. Improving wellbeing and mental health. (2023). Available online at: https://www.merck-animal-health-usa.com/offload-downloads/2023-vet-wellbeing-presentation (Accessed October 21, 2024).

[21] American Veterinary Medical Association, 2024 AVMA report on the economic state of the veterinary profession, (2024) Schaumburg, IL.

[22] American Veterinary Medical Association, 2022 AVMA report on the economic state of the veterinary profession, (2022) Schaumburg, IL.

[23] Reddy M. Small business in small economies: constraints and opportunities for growth. Inst. Soc. Econ. Stud. (2007): 304–321.

[24] American Express. American Express reveals 13th annual small business Saturday® encourages consumers to shop small® throughout the holiday season and beyond. (2022). Available online at: https://www.americanexpress.com/en-us/newsroom/articles/regions/american-express-reveals-13th-annual-small-business-saturday.html (Accessed October 24, 2024).

[25] Mars Veterinary Health. Veterinary sustainability. (2025). Available online at: https://marsveterinary.com/veterinary-sustainability/ (Accessed March 31, 2025).

[26] NVA Newsroom. ‘NVA announces $10 million donation to fund clinical trials and pioneering breakthroughs in specialty and emergency medicine’. (2025). Available online at: https://www.nva.com/newsroom/nva-announces-10-million-donation-to-fund-clinical-trials-and-pioneering-breakthroughs-in-specialty-and-emergency-medicine (Accessed March 31, 2025).

[27] Osborne D. The corporatization of veterinary medicine. Can Vet J. (2023) 64:483–8. PMID: 37138718 PMC10150558

[28] Stigler GJ. The economies of scale. J Law Econ. (1958) 1:54–71. doi: 10.1086/466541

[29] Gabor S Danylenko G Voegeli B. Familiarity with artificial intelligence drives optimism and adoption among veterinary professionals: 2024 survey. Am J Vet Res. (2025) 86:S63–9. doi: 10.2460/ajvr.24.10.0293, PMID: 39933254

[30] American Veterinary Medical Association, 2025 AVMA report on the economic state of the veterinary profession, (2023) Schaumburg, IL.

[31] Shock DA Roche SM Genore R Renaud DL. The economic impact that registered veterinary technicians have on Ontario veterinary practices. Can Vet J. (2020) 61:505–11. PMID: 32355349 PMC7155880

[32] American Veterinary Medical Association. Utilizing veterinary technicians to improve practice success. (2024). Available online at: https://www.avma.org/resources-tools/practice-management/utilizing-veterinary-technicians-improve-practice-success (Accessed September 24, 2024).

[33] Ouedraogo FB Lefebvre SL Salois M. Nonveterinarian staff increase revenue and improve veterinarian productivity in mixed and companion animal veterinary practices in the United States. J Am Vet Med Assoc. (2022) 260:916–22. doi: 10.2460/javma.21.11.0482, PMID: 35333739

[34] Mattson K. Valuing veterinary technicians in practice. J Am Vet Med Assoc. (2020) 257:778–9.

[35] Ouedraogo FB Weinstein P Lefebvre SL. Increased efficiency could lessen the need for more staff in companion animal practice. J Am Vet Med Assoc. (2023) 261:1357–62. doi: 10.2460/javma.23.03.0163, PMID: 37257829

[36] Boursiquot N Prendergast H Boudreau L Cital SN Mages A Rauscher J. (2023) 2023 AAHA Technician Utilization Guidelines. American Animal Hospital Association. Available online at: https://www.aaha.org/resources/2023-aaha-technician-utilization-guidelines/ (Accessed January 8, 2025).

[37] Gitter R LaFayette B. (2024). Demand for and supply of veterinarians in the US to 2032. Available online at: https://www.aavmc.org/wp-content/uploads/2024/06/Demand-for-and-Supply-of-Veterinarians-in-the-U.S.-to-2032-New.pdf (Accessed March 30, 2025).

[38] Kogan LR Stewart SM. Veterinary professional associates: does the profession’s foresight include a mid-tier professional similar to physician assistants? J Vet Med Educ. (2009) 36:220–5. doi: 10.3138/jvme.36.2.220, PMID: 19625672

[39] Larkin M. A proliferation of newly proposed veterinary colleges: universities from South Carolina to Utah have lined up to seek accreditation from the AVMA Council on education. J Am Vet Med Assoc. (2023) 261:1613–5.

[40] Larkin M. US veterinary colleges increase seats at accelerating rate: more than a third of institutions have had double-digit increases in first-year seats in the past five years. J Am Vet Med Assoc. (2023) 261:1611–2.

[41] MacLachlan MJ Volk J Doherty C. Incorporating model selection and uncertainty into forecasts of economic conditions in companion animal clinical veterinarian labor markets. J Am Vet Med Assoc. (2024) 1:1–8. doi: 10.2460/javma.24.09.0624 39586176

[42] American Veterinary Medical Association. Veterinarian’s oath. (2024) Available online at: https://www.avma.org/resources-tools/avma-policies/veterinarians-oath (Accessed October 24, 2024).

[43] Bart CK Bontis N Taggar S. A model of the impact of mission statements on firm performance. Manag Decis. (2001) 39:19–35. doi: 10.1108/EUM0000000005404

[44] JAB Holding Company. Our responsibility. (2024). Available online at: https://www.jabholco.com/our-responsibility (Accessed January 22, 2024).

[45] Loyal Family Veterinary Hospital. Available online at: https://www.loyalfamilyvet.com. (2024). (Accessed January 22, 2024)

[46] Cornell University College of Veterinary Medicine. The Center for Veterinary Business and Entrepreneurship. (2024). Available online at: https://www.vet.cornell.edu/departments-centers-and-institutes/center-veterinary-business-and-entrepreneurship (Accessed January 23, 2024).

[47] Veterinary Management Groups. Find your peer group. Find your future. (2025). Available online at: https://www.myvmg.com/membership/intellectual-capital/ (Accessed 31 March 2025).

[48] Neill C Salois M. Data on file VMG Economic Update. John’s Creek, Georgia:VMG data on file. (2024).

[49] Elce Y. The mentor-mentee relationship, addressing challenges in veterinary medicine together. Vet Clin Small Animal Pract. (2021) 51:1099–109. doi: 10.1016/j.cvsm.2021.04.023, PMID: 34238600

[50] Freeman D Hodgson K Darling M. Can mentorship improve the transition from veterinary school to clinical practice? J Am Vet Med Assoc. (2022) 260:1620–4. doi: 10.2460/javma.22.06.0249, PMID: 35947683

[51] Niehoff BP Chenoweth P Rutti R. Mentoring within the veterinary medical profession: veterinarians’ experiences as protégés in mentoring relationships. J Vet Med Edu. (2005) 32:264–71. doi: 10.3138/jvme.32.2.264 16078181

[52] Yanke AB Weigand KA Hofmeister EH. Addressing the needs and challenges of mentorship in veterinary medicine. New Dir Teach Learn. (2023) 2023:83–93. doi: 10.1002/tl.20571

[53] Suveto. Veterinarians. (2024). Available online at: https://suveto.com/veterinary-ownership/ (Accessed April 13, 2024).

[54] Independent Veterinary Practitioners Association. (2024) Available online at: https://www.iveterinarians.org/ (Accessed April 13, 2024).

[55] Veterinary Hospital Management Association. Group purchasing organizations in veterinary medicine. (2020). Available online at: https://www.vhma.org/blogs/vhma-admin/2020/09/25/group-purchasing-organizations (Accessed October 24, 2024).

[56] Taneja S Pryor MG Hayek M. Leaping innovation barriers to small business longevity. J Bus Strategy. (2016) 37:44–51. doi: 10.1108/JBS-12-2014-0145

[57] United States Fedeyral Trade Commission Decision and order docket C-4770, (2022).

[58] The White House. The importance of competition for the American economy. (2021). Available online at: https://bidenwhitehouse.archives.gov/cea/written-materials/2021/07/09/the-importance-of-competition-for-the-american-economy/ (Accessed November 22, 2024).

[59] Social Market Foundation. Consumers and the economy are getting a bad deal because companies don’t face enough competition. (2017). Available online at: https://www.smf.co.uk/consumers-economy-getting-bad-deal-companies-dont-face-enough-competition-event/ (Accessed November 22, 2024).

[60] Department of Justice. Competition and monopoly: Single-firm conduct under section 2 of the Sherman act: Chapter 2. (2022). Available online at: https://www.justice.gov/archives/atr/competition-and-monopoly-single-firm-conduct-under-section-2-sherman-act-chapter-2#:~:text=Introduction,case%20in%20a%20competitive%20market.

[61] Haucap J Stiebale J. Research: innovation suffers when drug companies merge. Harvard Business Review (2016). Available online at: https://hbr.org/2016/08/research-innovation-suffers-when-drug-companies-merge (Accessed November 22, 2024).

[62] Neill C Salois M. Data on file: Survey of former practice owners. GA, United States: Veterinary Management Groups (2023).

[63] Brakke Consulting. Industry overview. Orlando, FL: Brakke Consulting (2023).

[64] U.S. Securities and exchange commission. Private funds. (2024). Available online at: https://www.sec.gov/resources-small-businesses/capital-raising-building-blocks/private-funds#:~:text=Private%20funds%20are%20not%20required,cannot%20publicly%20offer%20its%20securities. (Accessed November 22, 2024).

[65] Ewens M Farre-Mensa J. The deregulation of the private equity markets and the decline in IPOs. Rev Financ Stud. (2020) 33:5463–509. doi: 10.1093/rfs/hhaa053

[66] Hamarat Ç Broby D. Regulatory constraint and small business lending: do innovative peer-to-peer lenders have an advantage? Financ Innov. (2022) 8:73. doi: 10.1186/s40854-022-00377-y

[67] Jones MD Harbin T. The business side of veterinary medicine: What veterinary schools don’t teach you. Maitland, FL, US: Vetbooks (2017).

